# Predictors of human error among intensive care unit nurses: the roles of professional commitment and anxiety

**DOI:** 10.3934/publichealth.2026020

**Published:** 2026-03-26

**Authors:** Aziza Mohammed Salem, Ahmad Mahmoud Saleh

**Affiliations:** 1 Nursing Department, Umluj College, Tabuk, University of Tabuk, Saudi Arabia; 2 College of Nursing, Prince Sattam Bin Abdulaziz University, Al-Kharj 11942, Kingdom of Saudi Arabia

**Keywords:** professional commitment, anxiety, human errors, intensive care unit, nurses, sustainable development goals

## Abstract

**Introduction:**

The intensive care unit (ICU) is a stressful environment where anxiety can increase human errors, affecting patient safety. Professional commitment can improve care quality and reduce such errors. In alignment with SDG 3 (Good Health and Well-being) and SDG 8 (Decent Work and Economic Growth), we explored the relationship between professional commitment, anxiety, and human errors among ICU nurses in two government hospitals in Riyadh and Tabuk, Saudi Arabia.

**Methods:**

This descriptive cross-sectional study was conducted among ICU nurses in two governmental hospitals in Riyadh and Tabuk, Saudi Arabia. Convenience sampling was used. Data were collected using a demographic questionnaire, the Spielberger Trait Anxiety Questionnaire, a professional commitment scale, and a researcher-made questionnaire on human errors in the ICU. Statistical analysis was performed using SPSS software, applying descriptive statistics, the Pearson correlation coefficient, and multiple linear regression.

**Results:**

A significant negative relationship was found between professional commitment and human errors, and a significant positive relationship between anxiety and human errors among ICU nurses (r = −0.148, p = 0.015; r = 0.174, p = 0.006, respectively). A multiple linear regression model containing both predictors was significant [F(2,151) = 9.84, p < 0.001] and explained 11.5% of the variance in human error scores (R² = 0.115).

**Conclusion:**

There is a significant association between professional commitment, anxiety, and human errors among ICU nurses. Reducing anxiety and improving professional commitment can help minimize human errors, improve patient safety, and contribute to the achievement of SDG 3 and SDG 8.

## 1. Introduction

Intensive care units (ICUs) are highly stressful and demanding environments where effective cooperation among the members of the healthcare team is essential to ensure patient safety [1]. However, achieving this coordination can be challenging. Inappropriate emotional responses, such as anxiety, can significantly affect the performance of individuals, leading to mistakes and adverse events. Stress and anxiety are common experiences for healthcare workers, often intensified by high workloads, critically ill patients, and the need for rapid decision making [2]. Although these pressures may be manageable in the short term, prolonged exposure can lead to emotional exhaustion and reduced efficiency. Due to the continued presence of stressors in hospital settings, especially critical areas like the ICU, healthcare professionals face higher levels of stress compared to other work sectors. Furthermore, persistent anxiety can negatively affect work performance and decrease overall productivity [1],[2].

Anxiety is defined as a diffuse and unpleasant feeling of worry accompanied by a sense of uncertainty in interpreting important events. It can arise from biological, environmental, and genetic factors [2]. Studies have shown that increased anxiety, especially during the COVID-19 pandemic, has been associated with a rise in medication and clinical errors among healthcare workers. In ICUs, where patient conditions are critical and workload is high, anxiety can significantly compromise performance and safety [3],[4]. Ensuring patient safety, defined as preventing harm resulting from healthcare errors or negligence, remains a fundamental priority in nursing practice. Evidence suggests that most incidents threatening patient safety are related to human error [5].

Human error in healthcare refers to the failure to perform a task or an error in planning an action that leads to patient harm, prolonged hospitalization, long-term disability, or death [6]. In ICUs, the high stress and complexity of nurses' responsibilities are major contributors to such errors [7]. Studies indicate that medical errors account for a substantial number of preventable deaths around the world. For example, research has shown that a significant proportion of nurses report involvement in at least one error in a short period, which can compromise patient safety, cause injuries, and reduce trust in healthcare systems [8],[9].

In this regard, professional commitment is an important factor that influences the qualifications and performance of healthcare providers. Professional commitment can be defined as a mental state reflecting an individual's desire and commitment to remain engaged in a profession [10]. Studies have shown that nurses with higher professional commitment demonstrate a stronger sense of responsibility, adherence to professional standards, and commitment to patient care, which can improve safety and quality in ICUs [10],[11]. Recognizing the importance of addressing human errors and the factors that contribute to them, and given the limited research in this area, we aimed to examine the relationship between professional commitment, anxiety, and human errors among nurses in the ICU.

Conceptually, professional commitment and anxiety may exert opposing influences on clinical performance and error risk. Higher professional commitment, which encompasses emotional attachment, a sense of obligation, and perceived investment in the nursing role, is theorized to promote behaviors that protect against errors. These include increased vigilance, meticulous adherence to safety protocols, and greater personal accountability for patient outcomes [10],[11]. In contrast, elevated anxiety, particularly the trait anxiety prevalent in high-stress environments such as the ICU, is hypothesized to affect cognitive function. It can narrow perceptual focus, hinder decision making, and reduce working memory capacity, thus increasing susceptibility to mistakes [3],[4]. Therefore, we posit that anxiety acts as a risk factor for human error, while professional commitment acts as a protective factor. Examining its concurrent relationship with errors provides a more comprehensive model of psychological determinants of safety in critical care nursing.

To address our objectives of this study, a descriptive cross-sectional study was conducted, as detailed in the following section.

## 2. Methods

### 2.1. Study design and participants

Ethical approval was obtained from the Standing Committee for Bioethics in the Deanship of Scientific Research (SCBR–035–2022), and subsequent administrative permissions were obtained from the nursing directors of two governmental hospitals located in Riyadh and Tabuk, Saudi Arabia. This cross-sectional study was conducted between January and March 2023 in government hospitals in the Kingdom of Saudi Arabia. The study population included all nurses from the ICU working in these hospitals. Participants were selected using a convenience sampling method.

The required sample size for this study was calculated using G*Power software [12] for a multiple regression analysis (fixed model, R² deviation from zero), with a significance level of 0.05, a power (1-β) of 0.80, 2 independent variables in the final model, and a small to medium anticipated effect size (f² = 0.10). This calculation indicated a minimum sample of 123 participants. To account for potential non-response, the target was increased to 154 ICU nurses. Participants were selected using a convenience sampling method from governmental hospitals in the Kingdom of Saudi Arabia. Inclusion criteria required a minimum of experience in the six months of ICU and willingness to participate, while incomplete questionnaires were excluded from the study.

A total of 180 questionnaires were distributed to all eligible ICU nurses in the two hospitals. Of these, 154 were completed and returned, yielding a response rate of 85.6%. Incomplete questionnaires (n = 26) were excluded according to the predefined criteria.

### 2.2. Data collection

After obtaining ethical approval and the necessary permissions, the researcher collected data from ICUs in two governmental hospitals in Riyadh and Tabuk, Kingdom of Saudi Arabia. After introducing the study and its objectives, participants were recruited using a convenience sampling method. ICU nurses who met the inclusion criteria provided their informed consent before participating. Each participant completed a four-part questionnaire that included a demographic survey, a professional commitment scale, the Spielberger trait anxiety questionnaire, and a human error questionnaire. To maximize participation and ensure accurate responses, data collection was performed in different work shifts (morning, afternoon, and night) and during breaks, giving nurses enough time to complete the questionnaires without disrupting patient care.

### 2.3. Measurement tools

#### 2.3.1. Demographic questionnaire

This questionnaire collected demographic information from participants, including age, gender, education level, marital status, and years of work experience.

#### 2.3.2. Professional commitment questionnaire

Professional commitment was assessed using the Mayer, Allen, and Smith [13] scale, comprising 18 items in three dimensions: affective, normative, and continuance commitment. The responses were rated on a 5-point Likert scale (1 = strongly disagree to 5 = strongly agree), yielding total scores ranging from 18 to 90. Scores below 30 indicated low professional commitment, 31–60 indicated moderate commitment, and scores above 61 indicated high commitment. Studies have demonstrated strong reliability for this instrument. For example, in a study conducted by Efthymiopoulos and Goula (2024), Cronbach's alpha coefficients for affective, continuance, and normative commitment dimensions were 0.87, 0.75, and 0.79, respectively [14]. In this study, the validity of the face and content was confirmed by experts, and reliability was evaluated using Cronbach's alpha: affective commitment = 0.52, continuance commitment = 0.72, normative commitment = 0.70, and overall instrument = 0.81.

#### 2.3.3. Trait anxiety inventory

Anxiety was assessed using the 20-item Spielberger Trait Anxiety Inventory (STAI-Y) [15]. Each item was rated on a 4-point Likert scale (1 = very little, 2 = little, 3 = a lot, 4 = very much). For the anxiety items, scores for statements 1, 3, 6, 7, 10, 13, 14, 16, and 19 were reverse-coded during the analysis. Total scores ranged from 20 to 80, with 20 to 30 indicating no anxiety, 31 to 42 mild anxiety, 43 to 53 moderate anxiety, and 54 to 80 severe anxiety. STAI-Y has shown high internal consistency, with Cronbach's alpha coefficients of 0.90 for trait anxiety [16].

#### 2.3.4. Human errors in the ICU questionnaire

A questionnaire was designed for this study to assess human errors among ICU nurses. The initial items were generated through interviews with ICU nurses and a review of the literature. To evaluate the psychometric properties, validity and reliability were assessed. For qualitative face validity, 15 ICU nurses reviewed the items for clarity, relevance, and appropriateness. Quantitative face validity was determined by calculating the impact score (IS) for each item based on nurses' ratings. An IS value greater than 1.5 was considered acceptable, indicating that the item was deemed relevant and appropriate by the participants [17]. Qualitative content validity involved 15 experts and faculty members who provided feedback on grammar, wording, item placement, and scoring. Quantitative content validity was assessed using the content validity ratio (CVR) and the content validity index (CVI). For a panel of 15 experts, a CVR value greater than 0.49 was considered acceptable and a CVI above 0.79 indicated strong content validity. Reliability was tested on a sample of 30 nurses, yielding a Cronbach's alpha of 0.87, indicating high internal consistency and confirming the reliability of the questionnaire.

### 2.4. Statistical analysis

Quantitative data was described using mean and standard deviation, while qualitative data were summarized using frequency and percentage. To examine the relationship between outcome variables and demographic characteristics, an independent two-sample t-test was used to compare means between variables with two categories (e.g., gender), and one-way analysis of variance (ANOVA) with post hoc pairwise comparisons was used for variables with three or more categories (e.g., education level). For the major study objectives, correlations between quantitative variables were assessed using the Pearson correlation coefficient. To evaluate the predictive role of anxiety and professional commitment on human errors, a multiple linear regression analysis was performed, entering both predictors simultaneously into the model to examine their unique contributions. The normality of the data was evaluated using the Shapiro-Wilk test. All statistical analyzes were performed using SPSS software version 25 [18], with a significance level set at p < 0.05. The results of the Shapiro-Wilk test were as follows: Total professional commitment (W = 0.987, p = 0.152), Anxiety (W = 0.991, p = 0.341), and human errors (W = 0.985, p = 0.089), supporting the assumption of normality. The multicollinearity among predictors was assessed using the variance inflation factor (VIF) and tolerance statistics. The characteristics of the study sample and the results of the statistical analyzes are presented below.

### 2.5. Ethical considerations

Permission to use the study instruments was obtained from the respective authors. Ethical approval was obtained from the Standing Committee on Bioethics at the Deanship of Scientific Research (SCBR–035–2022), and additional approval was obtained from the governmental hospitals prior to data collection. All participating ICU nurses were informed about the study objectives and provided their written consent. Measures were taken to protect the confidentiality of the data collected, and participants were assured that they could withdraw at any time. The questionnaires and other study materials were coded to maintain anonymity, and all procedures were conducted in accordance with the ethical principles outlined in the Declaration of Helsinki.

## 3. Results

Most of participants were female (63%), 20–40 years (78.6%), and married (69.5%). Most had a bachelor's degree (75.3%), and more than half had 6–10 years of work experience. Further demographic details are presented in Table 1. One-way ANOVA revealed a significant relationship between age [F(2,151) = 6.45, p = 0.003] and education level [F(2,151) = 3.65, p = 0.031] with mean professional commitment scores. Post hoc analysis indicated that nurses who held an associate degree had significantly higher commitment scores than those who held a bachelor's degree. Additionally, the level of education was significantly correlated with anxiety scores (p = 0.006), and nurses with a bachelor's degree reproted the highest mean anxiety. Age was also significantly associated with human error scores (p = 0.001), with the highest mean error scores observed among nurses aged 20–40 years. Additional details are provided in Table 1.

**Table 1.** Distribution of demographic variables and their relationship with mean anxiety, professional commitment, and human errors among ICU nurses (n = 154).

Demographic variables	n (%)	Anxiety M (SD)	Anxiety	Professional commitment M (SD)	Professional commitment	Human Errors M (SD)	Human errors
Age
20–40	121 (78.6%)	46.2 (7.9)	0.69*	66.1 (9.8)	0.003 *	29.1 (7.2)	0.001*
41–60	28 (18.2%)	44.7 (7.5)		58.4 (10.6)		24.6 (6.5)	
Above 60	5 (3.2%)	43.1 (6.4)		60.2 (9.2)		22.4 (5.8)	
Gender
Female	97 (63.0%)	46.0 (8.1)	0.13**	63.9 (10.5)	0.21**	27.8 (7.3)	0.10**
Male	57 (37.0%)	44.2 (7.6)		62.3 (11.1)		26.2 (6.8)	
Marital status
Married	107 (69.5%)	45.6 (8.0)	0.82**	63.6 (10.4)	0.11**	27.1 (7.1)	0.22**
Single	47 (30.5%)	45.0 (7.8)		62.9 (11.2)		27.4 (7.5)	
Education level
Associate degree	21 (13.6%)	42.1 (6.6)	0.006*	69.2 (9.0)	0.031*	26.5 (6.3)	0.08*
Bachelor's degree	116 (75.3%)	47.1 (8.0)		62.0 (10.9)		27.9 (7.4)	
Master's degree and higher	17 (11.1%)	43.5 (7.2)		64.4 (10.1)		25.8 (6.1)	
Work experience
6–10 years	85 (55.2%)	46.0 (8.0)	0.32*	63.1 (10.5)	0.04*	28.3 (7.2)	0.13*
10–20 years	60 (39%)	44.6 (7.6)		64.6 (10.8)		26.6 (6.8)	
Above 20 years	9 (5.8%)	42.8 (6.7)		61.2 (11.0)		24.2 (6.0)	

Note: *p* < 0.05, **p* < 0.01. Significance tests: * = one-way ANOVA; ** = independent samples t test.

As shown in Table 2, the five most frequent human errors reported by ICU nurses were related to communication and procedural verification (e.g., lack of proper communication during admission: 79.9%; not checking medication/NPO status: 76.6%), insufficient number of nursing staff (72.7%), not reporting previous medical or surgical history to the physician (72.7%), and failure to maintain aseptic or infection control standards (70.1%). These findings indicate that communication failures, inadequate staffing, and procedural verification errors are among the most common challenges contributing to human error in ICUs. Such issues may be due to workload pressures, frequent interruptions, and communication barriers between members of the healthcare team.

On the contrary, the five least frequent human errors identified in this study were: Making the wrong decision under stress (52.0%), distractions during patient care (53.3%), quick decision making under pressure (55.2%), misjudging patient condition or clinical situation (60.4%), and lack of familiarity with the procedure or care protocol (62.3%) (Table 2). Although these errors occurred less frequently, they are critical to patient safety, as they are closely related to cognitive overload and stress in high-pressure environments. Addressing these areas through continuous education, teamwork training, and simulation-based practice could help minimize errors and improve patient care quality in the ICU setting.

**Table 2.** Frequency of human errors related to Duties Performed by Intensive Care Unit (ICU) (n = 154).

Human errors in the ICU	Disagree (n, %)	Neutral (n, %)	Agree (n, %)
Lack of proper communication between the nurse and the patient during admission	12 (7.8%)	19 (12.3%)	123 (79.9%)
The patient was not checking his identification or wristband	17 (11.0%)	20 (13.0%)	117 (76.0%)
Not reviewing care and medication sheets before procedures	10 (6.5%)	32 (20.8%)	112 (72.7%)
No verifying blood tests, charts, or physician orders	15 (9.7%)	30 (19.5%)	109 (70.8%)
Not reporting previous medical or surgical history to the physician	14 (9.1%)	28 (18.2%)	112 (72.7%)
Not checking or reporting patient allergies	21 (13.6%)	26 (16.9%)	107 (69.5%)
Not assessing hygiene, presence of medical devices, or jewelry	22 (14.3%)	23 (14.9%)	109 (70.8%)
Not verifying consent form details and signatures	23 (14.9%)	21 (13.6%)	110 (71.4%)
Not checking medication intake or NPO status before procedures	16 (10.4%)	20 (13.0%)	118 (76.6%)
Uncertainty about the planned procedure or intervention	18 (11.7%)	25 (16.2%)	111 (72.1%)
Uncertainty about the affected side or site of procedure	14 (9.1%)	35 (22.7%)	105 (68.2%)
Improper equipment setup or placement	16 (10.4%)	47 (30.5%)	91 (59.1%)
Inappropriate patient positioning during care	21 (13.6%)	33 (21.4%)	100 (65.0%)
Incorrect counting or recording of medical items	28 (18.2%)	31 (20.1%)	95 (61.7%)
Failure to maintain aseptic or infection control standards	19 (12.3%)	27 (17.5%)	108 (70.1%)
Improper use of medical equipment	10 (6.5%)	38 (24.7%)	106 (68.8%)
Inadequate site preparation or disinfection before procedures	13 (8.4%)	35 (22.7%)	106 (68.8%)
Unawareness of patient allergies	20 (13.0%)	34 (22.1%)	100 (64.9%)
Failure to identify the correct patient	22 (14.3%)	24 (15.6%)	108 (70.1%)
Retained or misplaced medical materials or devices	23 (14.9%)	31 (20.1%)	100 (64.9%)
Lack of familiarity with the procedure or care protocol	18 (11.7%)	40 (26.0%)	96 (62.3%)
Quick decision-making under pressure	17 (11.0%)	52 (33.8%)	85 (55.2%)
Distractions during patient care	15 (9.7%)	57 (37.0%)	82 (53.3%)
Insufficient number of nursing staff	14 (9.1%)	28 (18.2%)	112 (72.7%)
Staff fatigue	11 (7.1%)	45 (29.2%)	98 (63.6%)
Lack of proper orientation or training for new staff	8 (5.2%)	50 (32.5%)	96 (62.3%)
Poor communication among ICU team members	18 (11.7%)	59 (38.3%)	77 (50.0%)
Performing multiple tasks simultaneously	13 (8.4%)	39 (25.3%)	102 (66.2%)
Loss of concentration during critical procedures	10 (6.5%)	46 (29.9%)	98 (63.6%)
Making the wrong decision under stress	7 (4.5%)	67 (43.5%)	80 (52.0%)
Misjudging patient condition or clinical situation	6 (3.9%)	55 (35.7%)	93 (60.4%)
Providing incorrect or incomplete patient information	9 (5.8%)	41 (26.6%)	104 (67.5%)

**Table 3.** Correlation between the mean scores of professional commitment and anxiety with human errors among ICU nurses (n = 154).

Independent Variable	Human Errors
Professional Commitment
Affective Commitment	r = −0.218, *p* = 0.002
Continuance Commitment	r = −0.158, *p* = 0.010
Normative Commitment	r = −0.075, *p* = 0.205
Total Professional Commitment Score	r = −0.148, *p* = 0.015
Anxiety
Total Anxiety Score	r = 0.174, *p* = 0.006

**Table 4.** Multiple linear regression analysis predicting human errors from anxiety and professional commitment (n = 154).

Independent Variable	B	SE B	β (Beta)	t	p-value	95% Confidence Interval for B
Constant	4.182	0.142	—	29.49	< 0.001	[3.90 – 4.46]
Anxiety	0.007	0.003	0.161	2.62	0.010	[0.002 – 0.012]
Professional Commitment	−0.004	0.001	−0.226	–3.74	< 0.001	[−0.006 – 0.002]

Note: B = unstandardized coefficient; SE B = standard error of B; β = standardized coefficient. VIF = Variance Inflation Factor (all values < 2).

As shown in Table 3, a significant negative correlation was found between human errors and affective (r = −0.218, p = 0.002) and continuance (r = −0.158, p = 0.010) components of professional commitment. Similarly, the total professional commitment score showed a modest yet significant negative relationship with human errors (r = −0.148, p = 0.015), suggesting that greater professional commitment is associated with fewer human errors among ICU nurses. On the contrary, a significant positive correlation was identified between anxiety and human errors (r = 0.174, p = 0.006), indicating that elevated anxiety levels can contribute to an increased probability of errors in intensive care settings. The detailed results of this analysis can be found in Table 3.

A multiple linear regression model with anxiety and professional commitment was tested as predictors of human error scores. The overall model was statistically significant, with F(2, 151) = 9.84, p < 0.001, and explained approximately 11.5% of the variance in human error scores (R² = 0.115, Adjusted R² = 0.104). Multicollinearity diagnostics indicated that there was no cause for concern, with all VIF values well below 2 (Anxiety: VIF = 1.02; Professional commitment: VIF = 1.02), confirming the independence of the predictors in the model. As shown in Table 4, both predictors made significant unique contributions. Anxiety had a positive and significant effect on human errors (B = 0.007, p = 0.010), indicating that higher levels of anxiety were associated with a greater probability of committing errors. On the contrary, professional commitment demonstrated a significant negative predictive effect (B = −0.004, p = 0.001), suggesting that nurses with stronger professional commitment were less likely to make errors during patient care. Together, these findings emphasize the dual importance of reducing anxiety and enhancing professional commitment to improve safety performance in intensive care settings. The following discussion interprets these findings in the context of the literature and the pressures of the ICU environment.

## 4. Discussion

We aimed to examine the relationship between anxiety, professional commitment, and the frequency of common human errors among nurses in the ICU working in two governmental hospitals, Kingdom of Saudi Arabia. A total of 154 ICU nurses participated in the study, most of them aged between 20 and 40 years.

We found that the average level of anxiety among nurses in the ICU was moderate. This finding aligns with previous research conducted in Saudi Arabia, where moderate levels of anxiety and stress were reported among critical care nurses [19]. Similarly, a study by Almutairi and El Mahalli [20] revealed that ICU nurses in Saudi hospitals frequently experience moderate anxiety due to the high workload and the high patient acuity. In terms of professional commitment, this study showed a moderate level among the participants, with the normative commitment scoring the highest. This is comparable to the findings of Alotaibi et al. [21], who reported that Saudi nurses demonstrated moderate to high levels of professional commitment, particularly those working in tertiary hospitals. However, these results differ from those of regional studies such as Mohamed et al. [22] in Egypt, which showed lower levels of commitment among nurses due to limited institutional support and high job stress. Differences between studies can be attributed to variations in work environments, resources, and opportunities for professional development available to nurses in different Arab countries.

Furthermore, our findings of this study revealed that the average level of human errors among nurses in the ICU was moderate. The errors reported the most frequently were related to “incomplete communication with patients during admission’ and “failure to check medication or treatment orders before procedures. These findings are consistent with those of Eltaybani et al. [23], who reported that communication-related errors were among the most common incidents in ICUs across hospitals. Similarly, a study by Mohamed et al. [22] in Egypt revealed that 34% of nursing errors were associated with inadequate patient evaluation and failure to verify medication orders. In another Saudi-based study, Alqahtani [24] emphasized that miscommunication during patient handover and document lapses were major contributing factors to human errors in critical care units. These results highlight the importance of improving communication and adherence to safety protocols during patient admission and handover processes in the ICU, as doing so may significantly reduce medical errors and improve patient safety.

Examining the relationship between the demographic characteristics of the ICU nurses in this study and their levels of anxiety revealed a significant association between educational level and anxiety. In particular, nurses holding associate degrees demonstrated the highest levels of anxiety. This finding contrasts with the results of Ali et al. [25], who examined job stress and anxiety among critical care nurses during the COVID-19 pandemic, Saudi Arabia, and found that nurses with higher educational qualifications reported higher anxiety levels. Similarly, Gan [26] observed that nurses with bachelor's degrees experienced greater stress due to increased professional responsibility and workload. The difference between this study and other findings may be attributed to contextual factors, such as variations in ICU staffing patterns, workload distribution, and institutional support systems after the pandemic.

An analysis of the relationship between the demographic characteristics of nurses in the ICU and their average human error scores revealed a significant correlation between age and error rates. In particular, the highest error scores were observed among nurses aged 20 to 40 years, possibly due to the demanding nature of ICU work and physical fatigue resulting from prolonged or intense activity. This finding aligns with the results of Alrasheeday et al. [27], who reported a direct and significant association between younger age and higher error rates among healthcare professionals in Saudi Arabia. On the contrary, a study by Tang et al. [28] involving 72 nurses indicated that error rates tend to decrease with age, contrasting with our findings. These discrepancies suggest that, in addition to age, other factors such as work experience, workload, and unit-specific stressors can influence the likelihood of errors among ICU nurses. Based on the results of this study, a direct and significant negative relationship was observed between professional commitment and human errors among the ICU nurses who participated. This finding aligns with the theoretical expectation that nurses with greater professional commitment, characterized by a stronger emotional attachment to their role (affective commitment), a deeper sense of moral obligation (normative commitment), and a higher perceived cost of leaving the profession (continuance commitment), are more likely to exhibit increased vigilance, stricter adherence to clinical protocols, and greater personal investment in patient safety outcomes. This increased sense of responsibility and engagement can act as a protective factor against lapses in attention and procedure, thereby reducing the probability of human error in the high-stakes ICU environment [10],[11]. The significant negative correlation with the affective and continuance commitment subscales underscores that emotional investment and perceived stakes in one's career are potent motivators for safe practice. On the contrary, earlier studies such as Baghaei et al. (2022) and Borrott et al. (2017) did not show a significant association between professional commitment and medication errors among nurses [29],[30]. The discrepancy may be due to differences in the types of errors studied, the settings of work, and the populations examined. On the contrary, Robat et al. (2020) investigated the relationship between professional commitment and knowledge, attitude, and practice of patient safety among nursing students and found that higher professional commitment improved patient safety performance [31]. Similarly, regional studies in Saudi Arabia, such as Alrashedi et al. (2021), reported that ICU nurses with greater professional commitment demonstrated fewer errors and improved adherence to patient safety protocols [32]. These findings are consistent with the results of this study, highlighting the importance of encouraging professional commitment to reduce human errors in critical care settings.

Interestingly, while affective and continuance commitment were negatively correlated with errors, normative commitment did not show a significant relationship. This suggests that a nurse's sense of moral obligation or duty to the profession (normative commitment) may not be as directly protective against errors in the high-stress ICU environment as emotional attachment (affective commitment) or perceived cost of leaving (continuance commitment). This distinction highlights that different dimensions of commitment can influence clinical performance in various ways, warranting further investigation.

An analysis of the relationship between anxiety levels and human errors among ICU nurses revealed that those with higher anxiety scores were more likely to commit errors. This finding is consistent with the results of Alameri et al. (2024), who found that fatigue and poor sleep quality among nurses in high-acuity settings in Saudi Arabia were strongly related to poor performance and a greater likelihood of clinical mistakes [33]. Similarly, Salameh et al. (2024) reported that perceived stress and alarm fatigue among intensive care nurses in Palestine significantly increased the risk of attention lapses and procedural errors [34]. Furthermore, Alinejad et al. (2023) highlighted that occupational stress and reduced emotional resilience among Iranian nurses were key contributors to decreased job performance and higher error rates [35]. Collectively, these studies reinforce our findings, emphasizing that unmanaged anxiety and stress can adversely affect nurses' concentration and decision making, ultimately increasing the risk of human error and compromising patient safety in intensive care environments.

## 5. Limitations

This study has several limitations. First, while data were collected from two regions (Riyadh and Tabuk), the use of convenience sampling limited the broader generalizability of the findings to other geographic, cultural, or healthcare settings within Saudi Arabia and internationally. The experiences and error patterns of ICU nurses may differ in other regions or countries with varying workloads, resources, institutional cultures, and nursing education systems. Therefore, future multicenter, multiregional studies employing random sampling methods are recommended to validate and extend these findings nationally and internationally. Second, the data was collected using a self-administered questionnaire, which may have introduced response bias. Therefore, researchers are encouraged to employ more objective tools, such as validated observational checklists or performance-based assessments. Furthermore, factors such as fatigue and workload may have affected the precision of the responses of the participants. To minimize this limitation, data collection in this study was carefully scheduled during less demanding shifts and nurses' rest periods. Furthermore, the internal consistency of the Affective Commitment subscale was low (α = 0.52), which may limit the reliability of the conclusions drawn for this component of professional commitment. In future studies, researchers should consider the use of scales with validated psychometric properties in similar populations.

## 6. Conclusion

Based on our findings in this study, there was a significant association between professional commitment and human errors, as well as between anxiety and human errors among ICU nurses. Additionally, professional commitment and anxiety were identified as predictors of human error risk in the intensive care setting. Therefore, implementing targeted interventions aimed at reducing anxiety levels and improving professional commitment may effectively minimize the occurrence of human errors and improve patient safety among ICU nurses.

## Use of AI tools declaration

The authors declare they have not used Artificial Intelligence (AI) tools in the creation of this article.

## Funding

This project was supported by the Deanship of Scientific Research at Prince Sattam Bin Abdulaziz University under the research project number (PSAU/2025/03/35024).
